# Functional Diversity of Microbial Communities in the Soybean (*Glycine max* L.) Rhizosphere from Free State, South Africa

**DOI:** 10.3390/ijms23169422

**Published:** 2022-08-20

**Authors:** Titilope Tinu Ajiboye, Ayansina Segun Ayangbenro, Olubukola Oluranti Babalola

**Affiliations:** Food Security and Safety Focus Area, Faculty of Natural and Agricultural Sciences, North-West University, Private Bag X2046, Mmabatho 2735, South Africa

**Keywords:** microbial communities, plant-microbe interactions, rhizodeposition, SEED subsystem, shotgun metagenomics

## Abstract

The plant microbiome is involved in enhancing nutrient acquisition, plant growth, stress tolerance, and reducing chemical inputs. The identification of microbial functional diversity offers the chance to evaluate and engineer them for various agricultural processes. Using a shotgun metagenomics technique, this study examined the functional diversity and metabolic potentials of microbial communities in the rhizosphere of soybean genotype link 678. The dominant genera are *Geobacter*, *Nitrobacter*, *Burkholderia*, *Candidatus*, *Bradyrhizobium* and *Streptomyces*. Twenty-one functional categories were present, with fourteen of the functions being dominant in all samples. The dominant functions include carbohydrates, fatty acids, lipids and isoprenoids, amino acids and derivatives, sulfur metabolism, and nitrogen metabolism. A Kruskal–Wallis test was used to test samples’ diversity differences. There was a significant difference in the alpha diversity. ANOSIM was used to analyze the similarities of the samples and there were significant differences between the samples. Phosphorus had the highest contribution of 64.3% and was more prominent among the soil properties that influence the functional diversity of the samples. Given the functional groups reported in this study, soil characteristics impact the functional role of the rhizospheric microbiome of soybean.

## 1. Introduction

Leguminous plants, such as soybean (*Glycine max* L.), provide high-protein and high-oil minerals for human use. Because of its high-quality plant-based protein and oil content, soybean is one of the world’s most significant crops [1]. The crop is grown on around 6% of arable land and 50% of legume-growing areas around the world [2]. The United States of America, Brazil, and Argentina are the world’s top soybean producers [3]. Soybeans are grown in many African countries, especially in Sub-Saharan Africa (SSA), and the crop is one of the most prevalent legumes in the region [1,4]. Soybeans are used for various purposes, including non-food applications like biodiesel production as well as human and animal nutrition. Because of their agricultural relevance and propensity to create symbiotic relationships with rhizobia, legume plants have been used to study plant-microbe interactions in the rhizosphere [5].

Plants are associated with diverse microbial populations that are taxonomically organized [6]. These organisms interact with their host plants in a complex and dynamic way and the environment has a great influence on these interactions [6,7]. Within the soil system, rhizosphere, or immediate environs of the plant root are microbial hotspots regarded as one of the most active interfaces on the planet where various interactions occur [8]. Additionally, the mutual interaction between plants and microbiomes occurs in the region around the roots of soybean plants due to the high variation of microbes in the rhizosphere. The rhizosphere’s microbial community is part of a complex food web that relies on nutrients (mainly exudates) supplied by the plant to regulate microbial activity and diversity in the rhizosphere [9]. This community is an important component of sustainable agriculture because it reduces fertilizer and pesticide consumption [5]. This microbiome is critical for plant growth, nitrogen fixation via nodulation, and environmental stress protection [10].

Despite the increasing importance of the microbiome in plant health and development, leveraging microbial interactions and functions to improve plant resilience to biotic and abiotic stress remains a challenge [6]. To address these challenges and ensure sustainable crop production, an understanding of the functional role of the microbial communities that colonize the plant rhizosphere is required. The microbiome functional diversity will aid in the discovery of appropriate and improved methods for increasing plant yield, particularly in nutrient-deficient and semi-arid regions of the world, where chemical inputs are required to supplement plant growth [11]. Rhizosphere microbial communities have been widely investigated using both culture-dependent and culture-independent approaches due to their importance in plant growth and performance [5,12]. Advances in next-generation sequencing techniques by using amplicon and shotgun sequencing have enabled in-depth investigations of the rhizosphere microbial community.

Metagenomics approaches have simplified the process of taxonomic and functional classification of uncultured microbial populations. This has facilitated a better understanding of microbial behavior and processes in their environments [11,13]. The technique has been used to obtain detailed information on the specific rhizosphere microorganisms [14], roles, number, and compositions [15,16]. In this study, a shotgun metagenomics approach, which enables the functional profiling of microbial communities inhabiting an environment, was used to unravel the microbial and functional diversity in the soybean rhizosphere. This is the foremost paper on the rhizosphere microbiome of the soybean genotype Link 678. The genotype is well adapted to cool and moderate areas of South Africa but susceptible to drought and root-knot nematode.

## 2. Results

### 2.1. Physicochemical Analysis of Soybean Soil

The results of soil properties are presented in Table 1. The soil samples were generally sandy, with a pH close to neutral. The rhizosphere samples (AA, CA, and AB) contained a uniform amount of clay contents and had lower clay contents compared to the bulk soil (BC). All samples contained a small amount of total carbon and nitrogen, organic carbon, and organic matter. The bulk soil was richer (BC) in carbon, organic matter, potassium, and total nitrogen than the rhizosphere samples. In contrast, the rhizosphere soils were richer in sulfur, phosphorus, and nitrogen in the form of ammonium (N-NH_4_^+^) than the bulk soil (Table 1). 

### 2.2. Sequence Processes

There were 5,090,756 sequences uploaded for sample AA, which contained 905,850,636 base pairs of 179 bp average length. Sample AB consisted of 5,684,005 sequences, with 973,653,572 bp at 175 bp average length, while sample CA contained 7,188,831 sequence counts, 1,114,054,964 bp at an average sequence length of 161 bp. The bulk soil (sample BC) was made up of 3,869,313 sequences, which contained 451,569,818 bp at an average length of 176 bp. After QC has been executed, the retained sequences in samples AA, AB, CA, and BC were 4,502,125, 5,042,523, 6,406,314, and 3,452,986 sequences, respectively. The predicted protein features in the samples were 4,007,282 for sample AA, 4,383,253 for sample AB, 5,716,457 for sample CA, and 3,045,886 for sample BC. Sample AA contained 2,337,594 (55.85%) unknown proteins, 2,698,188 (57.61%) and 3,468,264 (58.07%) unknown proteins were present in sample AB and CA, respectively, while bulk soil had 1,826,886 (57.18%) unknown proteins.

### 2.3. Taxonomy of the Microbiome in Both the Rhizosphere and Bulk Sample

**Figure 1:**
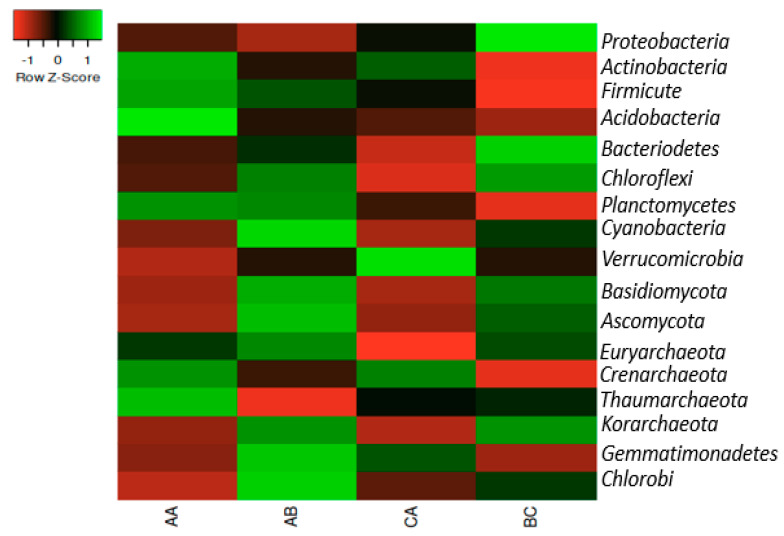
The heatmap representation of the relative abundances of the Phyla in the samples. The scale bar depicts a color saturation gradient based on relative abundances of the Phyla that have been z-score converted. AA, AB, and CA are rhizosphere soil samples while BC is a bulk soil sample.

The phyla in the soil samples were Proteobacteria, Actinobacteria, Firmicutes, Acidobacteria, Bacteroidetes, Chloroflexi, Planctomycetes, Cyanobacteria, Verrucomicrobia, Basidiomycota, Ascomycota, Euryarchaeota, Crenarchaeota, Thaumarchaeota, Korarchaeota, Gemmatimonadetes, and Chlorobi, but Proteobacteria predominates in rhizosphere samples. The phylum Actinobacteria predominates in the bulk soil. The most prominent archaeal is Euryarchaeota and this was dominant in all samples, while Korarchaeota was less dominant in both the rhizosphere and the bulk soil (Figure 1).

**Figure 2:**
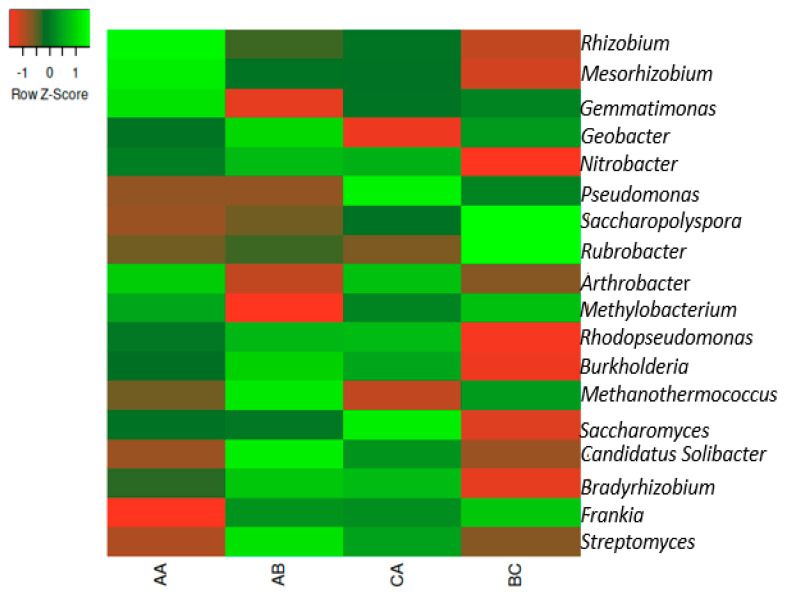
The heatmap representation of the relative abundances of the genera in the samples. The scale bar depicts a color saturation gradient based on relative abundances of the genera that have been z-score converted. AA, AB, and CA are rhizosphere soil samples while BC is bulk soil sample.

The following genera were found in all samples, as shown in Figure 2. *Bradyrhizobium*, *Streptomyces*, *Arthrobacter*, *Saccharopolyspora*, *Mesorhizobium*, *Nitrobacter*, *Geobacter*, *Rubrobacter*, *Frankia*, and *Burkholderia* were dominant in all samples (Figure 2). The distribution of the genera from various sampling sites is presented in Supplementary Figure S1. 

### 2.4. Functions of Microbiomes in the Soil 

**Figure 3:**
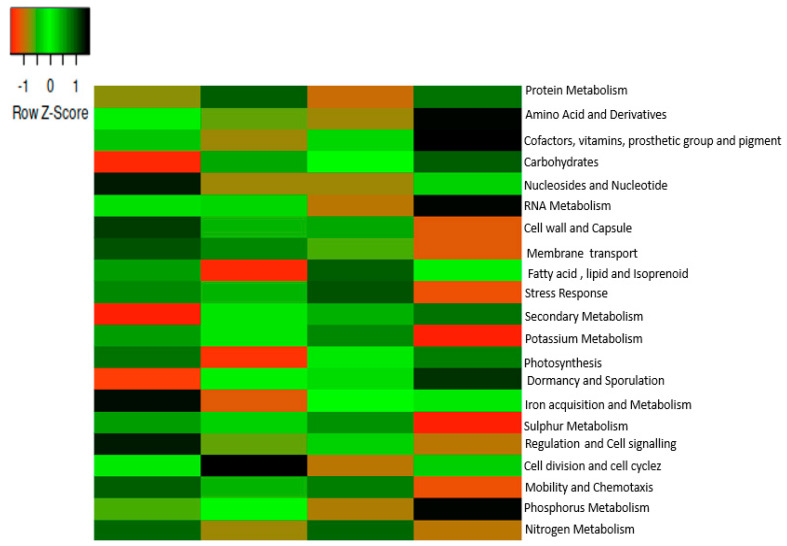
Metabolic functions at level 1 in the SEED subsystem. The scale bar depicts a color saturation gradient based on relative abundances of the functional categories that have been z-score converted. AA, AB, and CA are rhizosphere soil samples while BC is a bulk soil sample.

**Figure 4:**
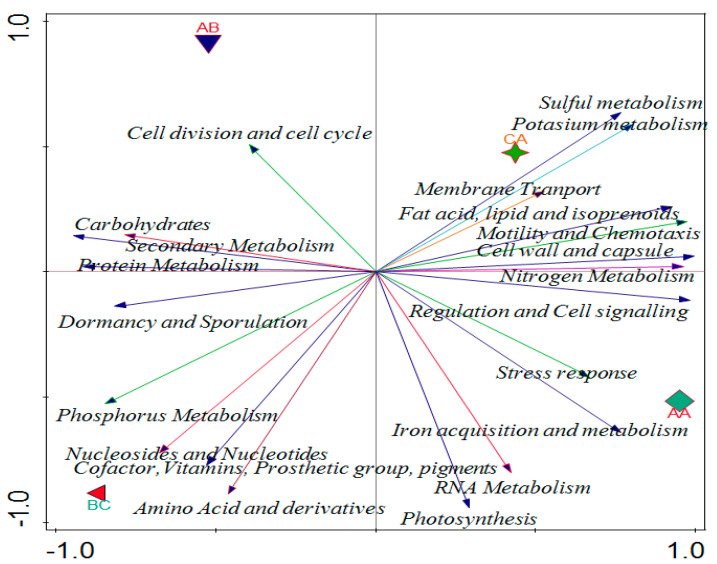
PCA analysis of functional categories of microbial communities in the rhizosphere and bulk soil samples showing the distribution of the microbial functions. AA, AB, and CA are rhizosphere soil samples while BC is a bulk soil sample.

The functions of the microbiomes were revealed at level 1 in all sampled soils with the help of SEED subsystem hierarchical gene annotation. Twenty-one functions were selected, as shown in Figure 3. Fourteen functions were prominent in samples AA and CA. The prominent functions were carbohydrates, vitamins, amino acids and derivatives, protein metabolism, cofactor, prosthetic groups, lipid, membrane transport, cell wall and capsule, RNA metabolism, motility and chemotaxis, and regulation and signaling. Twelve functions, such as carbohydrates, amino acid and derivatives, vitamins, protein metabolism, cofactor, prosthetic groups, pigments, lipid, membrane transport, cell wall and capsule, RNA metabolism, nucleoside and nucleotides, fatty acid, and isoprenoids, stress response, nitrogen metabolism, and phosphorus metabolism were prominent in sample AB and the bulk soil (sample BC). PCA (principal component analysis) was used to determine the functional categories distribution in both rhizosphere and bulk soils as shown in Figure 4. 

### 2.5. Microbiome Indices of Function in Soybean Soil Samples

Alpha diversity of the functional categories for microbiomes in soybean rhizosphere for level 1 was used to determine the evenness, Simpson’s and Shannon values (Table 2). The Shannon and Simpson’s indices were higher in the rhizosphere samples compared to the bulk soil sample. There are no significant differences obtained in the values in the diversity levels among the samples using the Kruskal–Wallis test at *p* > 0.05. The relative abundances of the functional categories within the subsystems at level 1 were visualized using the principal coordinate analysis (PCoA) plot (Figure S2), while the R- and *p*-values of 0.51 and 0.01, respectively, were obtained from the analysis of similarity (ANOSIM).

**Figure 5:**
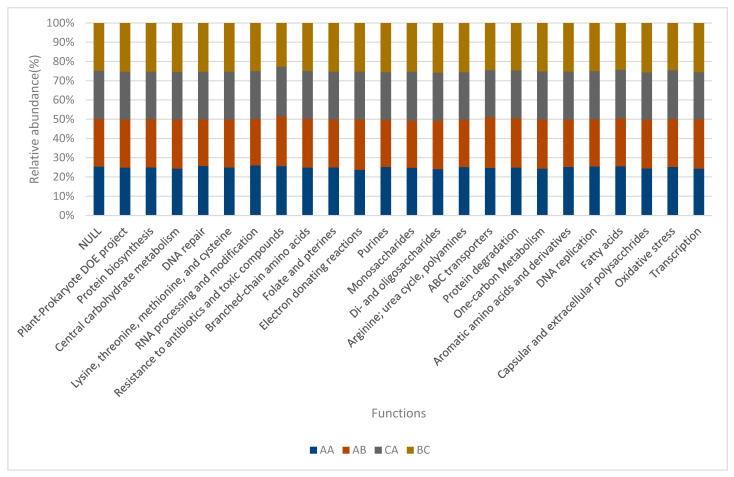
Bar-chart for selected functions in Level 2. AA, AB, and CA are rhizosphere soil samples while BC is a bulk soil sample.

The relative abundance of the functional categories in level 2 was used to develop a bar chart (Figure 5) with dominant functions based on the relative abundance. Relative abundance of all soil samples were close to one another in nearly all functions, but plant-prokaryote DOE project, protein biosynthesis, lysine, threonine, methionine, and cysteine (amino acid) of the bulk soil (BC) were more pronounced, although sample AB revealed high relative abundance values of central carbohydrate metabolism, while DNA repair of sample AA was more prominent than others.

### 2.6. Effects of Soil Properties on the Functional Categories of the Microbiome in the Soil Samples

**Figure 6:**
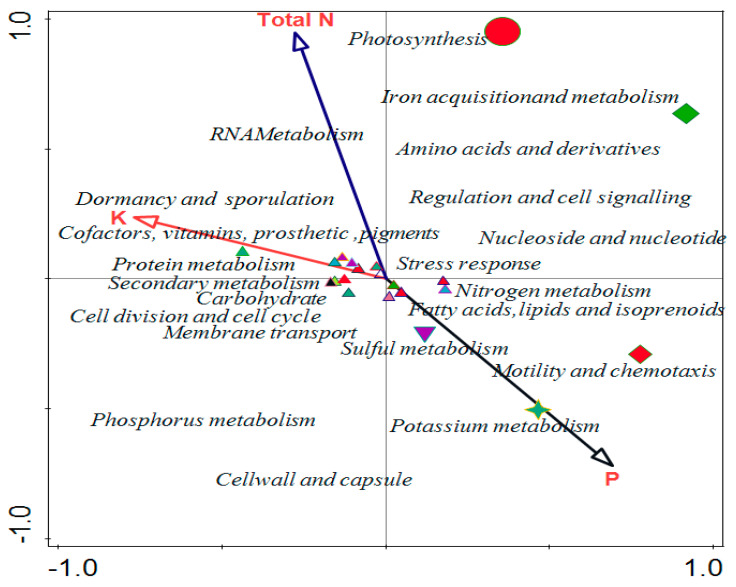
Canonical correspondence analysis showing the effect of physicochemical parameters on functional categories in the rhizosphere. AA, AB, and CA are rhizosphere soil samples while BC is a bulk soil sample.

Canonical correspondence analysis (CCA) was used to evaluate the relationship that exists between the soil properties of soybean and the functional levels of the microbial communities (Figure 6). Environmental conditions of the forward selection that are good for explaining variations showed phosphorus was more significant compared to other properties. Phosphorus has 0.718 *p*-values with a 64.3% percentage contribution in the variation, which is the same as the percentage explained (Figure 6). The contributions of other soil properties are shown in Table 3 and vector arrows (Figure 6) represent them.

### 2.7. Pathways Revealing the Functions of Microbiomes Living in the Soil Samples

The subsystem at level 3 revealed sequences involved in several pathways involved in fatty acid degradation regulons, serine glyoxylate cycle, DNA repair, inorganic sulphur assimilation, ammonia assimilation, bacterial chemotaxis, cobalt-zinc-cadmium resistance, and peptidoglycan biosynthesis (Figure S3). Fatty acid degradation regulons were more pronounced compared to other functions in sample AA, while the bulk soil sample had the least abundance (Figure S3). 

From the respiration (cell metabolism) pathway, terminal cytochrome C oxidases, formate dehydrogenase, and hydrogenases were more abundant in sample AB compared to other samples. The relative values of sample AB and bulk soil (BC) were higher (0.15%) in respiratory complex I, while samples AA and CA had the same values (0.14%). The relative abundance of 0.1% was obtained from F0F1-type ATP synthase, formate dehydrogenase, respiratory dehydrogenases 1, and anaerobic respiratory reductases in all samples. The highest value, 0.03%, was recorded in NiFe hydrogenase maturation for samples AB, CA, and bulk soil. The quinone oxidoreductase family, succinate dehydrogenase, and trim ethylamine N-oxide (TMAO) reductase were observed to have the same relative value of 0.01% in all soil samples. The value observed (0.004%) in the formate hydrogenase metabolic pathway in all rhizosphere samples was also recorded in samples AA and CA of V-Type ATP synthase metabolic pathway. However, the relative abundance of sample CA had its only highest value (0.003%) in the CO dehydrogenase metabolic pathway, with the lowest value (0.001%) from the bulk sample. The relative abundance (0.005%) of membrane bound hydrogenases metabolic pathway was highest in the bulk soil with the least value (0.003%) in samples AB and CA (Figure S4). 

Fatty acids, lipids, and isoprenoids pathways showed the same relative value of 0.1% in polyprenyl synthesis, glycerolipid and glycerophospholipid metabolism (Figure S5). Carotenoids metabolic pathways had the same relative abundance of 0.08% in all rhizosphere soil samples but it was more abundant in bulk soil. However, the bulk soil sample showed lower values in cholesterol catabolic operon and polyunsaturated fatty acids synthesis but the values of rhizosphere sample were the same for all samples. Sample AA showed the highest relative value (0.002%) in fatty acid biosynthesis FASII, while other samples had the same value of 0.001% (Figure S5).

The pathways needed in sulfur metabolism showed that alkanesulfonate utilization, thioredoxin-disulfide reductase and release of dimethyl sulphide (DMS) from dimethyl sulfonic propionate (DMSP) were 0.01%, 0.04% and 0.001% respectively, in all soil samples, both rhizosphere and bulk. The bulk soil sample showed the highest value (0.26%) in the inorganic sulfur assimilation pathway, while sample AA and CA were observed to have the least relative value (0.23%). However, it was observed that the DMSP breakdown pathway had the least value in sample AA, while sample BC (bulk soil) had the highest (Figure S6).

Nitrogen metabolism pathway unveiled common relative abundance values (0.01%) in the amidase clustered with urea and nitrile hydratase functions and nitrosative stress, but ammonia assimilation, denitrification, cyanate hydrolysis, and nitrilase (0.27%, 0.02%, 0.003%, and 0.001%, respectively) in all soil samples ((Figure S7). Samples AA and AB showed the same relative values of 0.15% in the nitric oxide synthase pathway with sample CA having the highest value of (0.16%). Nitrate and nitrite ammonification showed the highest value in samples AA and CA (0.12%) but the least in sample BA (0.10%). The lower relative values (0.03%) for allatioin utilization metabolism were revealed in the rhizosphere samples (AA, AB and CA). However, high nitrogen fixation was observed in the samples of the soil attached to the root (rhizosphere). Samples AA and AB had the highest relative value (0.003%) in CBSS-280355.3.peg.2835 while sample CA and bulk soil had the lowest values (0.002%).

In the metabolism pathway for amino acids in Figure S8**,** nine were selected from branched amino acids and ten from aromatic amino acids, histidine degradation, histidine biosynthesis, ketoisovalerate oxidoreductase, branched-chain amino acid biosynthesis, isoleucine degradation, branched chain amino acid degradation regulons, isoleucine degradation and HMG CoA metabolism, HMG CoA synthesis, valine degradation, isoleucine degradation, and leucine biosynthesis. A percentage of 0.1% was observed in all samples in the following pathways: ketoisovalerate oxidoreductase, branched-chain amino acid biosynthesis, isoleucine degradation, branched chain amino acid degradation regulons, histidine degradation, and branched chain amino acid degradation regulons, while 0.02% was observed in all samples for both aromatic amino acid degradation and isoleucine degradation.

## 3. Discussion

Plants influence rhizosphere microbial communities, which are crucial for crop growth and yield. The rhizosphere is home to a microbial community that plays a critical role in plant growth and productivity [17]. In this study, the functional profile of the microbial communities from the rhizosphere of soybean was examined. In this study, Proteobacteria, Acidobacteria, Bacteroidetes, and Actinobacteria were the dominant microbial community in soybean rhizosphere, which is consistent with earlier reports about the soybean rhizosphere microbiome [5,9]. Proteobacteria was consistently enriched in the rhizosphere of soybean in all samples. This agrees with the idea that copiotrophs, such as Proteobacteria and Actinobacteria, are abundant in nutrient-rich environments, such as the rhizosphere [18]. This indicates that the soybean rhizosphere is a rich environment for the growth and activity of the microbial group.

Sequence alignment to a curated database of annotated sequences is typically required for functional profiling of metagenomics sequences to find similar matches [19]. Functional investigation of the rhizosphere microbiome, along with compositional characterization, is necessary for understanding the microbiome assembly and boosting applications for sustainable agriculture, considering the functional redundancy between various microbial communities [20]. Alpha diversity from Shannon, Evenness and Simpson were obtained from the samples and was confirmed with a Kruskal–Wallis test with *p*-value 0.042. Index values for Shannon in all sample locations conformed with the 2.81 theoretical limits as established by Dinsdale et al. [21], but were lower than that reported by Li et al. [22]. Dominant functions were few in level 1 and caused the evenness indices to be lower. The dominant pattern’s difference showed the importance of some functions over others in the metagenomes. Beta diversity of the study was examined with PCoA using ANOSIM to establish the difference within the samples and composition results in 0.58 and 0.01 values for R and *p*-value, respectively. The differences in microbial functions between the rhizosphere and bulk samples showed the variations existed between the samples. The variation in cumulative value between functional categories were 72.55% and 94.04%, and Eigen values were 72.55% and 21.48% in axis 1 and 2, respectively (Figure 6). The variation in cumulative value between functional categories in this study was lower than the 73.2% reported by Kumar et al. [23]. A principal component analysis was used to test the variation between functions and metabolic potential in the samples. The total variation of 0.029%, explanatory variables of 47%, and adjusted explained variation of 20.50% show how omics can determine the metabolic function of the microbiomes in a particular sample.

Higher relative abundance was observed in the sequences associated with carbohydrate, amino acids and derivatives, protein metabolism, cofactor, vitamins, prosthetic group, pigments, DNA metabolism, and respiration in subsystem level 1. Carbohydrates are essential for energy and important for microbial survival. Soil microorganisms depend on various carbon sources for development and survival since they use carbon as a source of energy for their metabolism and growth. The abundance of functional categories related to carbon dioxide fixation, organic acids, sugar alcohols, amino sugars and glycoside hydrolases at level 2, and the presence of metabolic pathways for the serine glyoxylate cycle, isobutyryl-CoA to propionyl-CoA module, methylglyoxal metabolism, acetone butanol ethanol synthesis, and glycolysis and gluconeogenesis in the samples are evidence of carbon utilization from different sources. Mendes et al. and Dubey et al. reported a high relative abundance of carbohydrate in the soybean rhizosphere [9,24]. The findings showed that the microbial communities in our samples help plants acquire carbon through various metabolic pathways [25]. Amino acids are used by microbes as a source of energy for survival in nutrient-poor conditions and in soils with little organic matter [26]. The metabolism of amino acids and other nutrients is essential in symbiotic interactions that occur in the rhizosphere [27].

The process of fixing nitrogen in the symbiosis of soybean and rhizobia increases soybean yield [28]. In this study, the relative abundance of amino acid metabolism was relatively the same for the four samples, just as it has been reported by Liang et al. [29]. Higher nitrogen metabolism was noted in the rhizosphere samples compared with the bulk soil. This metabolism is regulated by Gram-positive bacteria in the nodules of legumes through bacteria fixation [30].

The microbes associated with nitrogen metabolism were observed at level 1. At level 3, ammonia assimilation, nitric oxide synthase, nitrate and nitrite ammonification, allantoin utilization, denitrification, nitrosative stress, amidase clustered with urea and nitrile hydratase functions, nitrogen fixation, cyanate hydrolysis, nitrilase, and dissimilatory nitrite reductase were observed. These metabolic pathways are essential for the formation of amino acids and protein metabolism.

Mineral elements, such as phosphorus, iron acquisition, potassium, and sulfur are essential for plant growth [31,32] and were enriched in all samples. As a result, our findings imply that the microbial communities present in the samples assist in the availability of crucial nutrients for the growth and development of soybean. All the samples show the dominance of these sulfur metabolic pathways: inorganic sulfur assimilation, galactosylceramide and sulfatide metabolism, alkanesulfonates utilization, sulfur oxidation, utilization of glutathione as a sulfur source, thioredoxin disulphide reductase, alkane sulfonate assimilation, taurine utilization, L-cystine uptake and metabolism, release of dimethyl sulphide from dimethyl sulfoniopropionate, and sulphate reduction-associated complexes. The numerous roles that microorganisms from various environments play in the enzymatic metabolism of sulfur have been described in previous studies [33,34]. This study shows that microbiomes can produce sulfur metabolic genes and protein enzymes, such as the sulfur carrier proteins adenylyl transferase ThiF, as cofactors to protect soybean plants during the biosynthesis of thiamine in the rhizosphere. Sulfate and thiosulfate import ATP-binding protein CysA (EC 3.6.3.25) protect soybean plants from stress response during the uptake of selenite. The findings in this study imply that the microbes living in the rhizosphere provide defense against oxidative stress [35]. 

The physicochemical results of soil samples showed that all samples contain sulfur in a good amount. We discovered that the chemical characteristics of the soil impacted on microbial functions using the CCA. Total N and K were positively correlated with RNA metabolism, dormancy and sporulation, and protein metabolism, while P positively correlated with nitrogen metabolism, fatty acids, lipids and isoprenoids, sulphur metabolism, and motility and chemotaxis. Potassium was identified as the soil parameter with the greatest influence on the composition of the microbial functional diversity. According to various studies, the characteristics of soils are the primary determinant of variation in the structural diversity of soil microbial communities [36], whereas the physical and chemical characteristics of soils also influence the functional diversity of microbial communities [37]. This study showed that the chemical properties of the soil played a role in determining the relative abundance of microbial functions in the sampling area.

## 4. Material and Method

### 4.1. Soil Sample Collection

In March 2021, the sample collection was done using a soil auger. Soybean rhizospheric soil samples were collected from a soybean farm in the Free-State Province of South Africa (27.28° S and 26.72° E) at a depth of 0–15 cm in triplicate as described by Chen et al. [38]. The bulk soil was sampled from a point that was approximately 10 m from the soybean field. Rhizosphere soil samples were collected after careful uprooting of the soybean plant at the fruiting stage. The rhizosphere soil samples were collected from three locations in the field and from each location, samples were collected in triplicates. The triplicate samples were labeled (AA, AB, and CA) and bulk soil (BC). The samples were placed in a sterile zip-lock bag, kept in a box containing ice packs, and taken to the laboratory. The samples were stored at −20 °C.

### 4.2. Physicochemical Analysis of the Soil Samples

The samples were ground and sieved using a 2 mm sieve and used for physicochemical analysis. Sáez-Plaza et al.’s [39] method was used to determine the total Nitrogen (N) level present in the soil. A pH meter was used to determine the pH of the samples in distilled water at a 1:2.5 soil to water ratio. The soil phosphorus was determined using Bray No. 1 solution as the extractant [40]. Based on the reaction with ammonium molybdate and the development of the “Molybdenum Blue” color, the extracted P was quantified colorimetrically. The amount of P removed from the soil was directly proportional to the compound’s absorbance measured at 882 nm with a spectrophotometer. Using 1 M KCl solution, the exchangeable N-NH_4_ and N-NO_3_ were determined as described by Kachurina et al. [41] and the absorbance was measured spectrophotometrically at 260 nm and 220 nm, respectively. Total N and C were determined using the dry combustion method as described by Wright and Bailey [42]. 

Ammonium acetate at a concentration of 1 mM with a neutral pH was used to determine the exchangeable potassium and sodium, while the sulfur was determined using HCl extraction. Organic matter was determined using the loss of ignition method described by Hoogsteen et al. [43]. The organic carbon was determined from the soil sample with the method explained by Walkley and Black [44].

### 4.3. Extraction of DNA and Sample Sequencing

The extraction of DNA was carried out from rhizosphere soil samples and the bulk soil using the DNeasy PowerSoil Pro kit (Qiagen, Hilden, Germany) following the instruction protocol. Samples were allowed to thaw; 0.25 g was weighed from each soil sample for DNA extraction. All the datasets used in this study are from the shotgun method of whole–metagenome sequencing. This was done at MR DNA, Shallowater, TX, USA. A life technologies assay kit-Qubit^®^ dsDNA HS was used to obtain samples’ DNA concentration. The libraries were prepared according to the manufacturer’s instructions using the Illumina DNA Prep (M) tagmentation library preparation kit. The libraries were made with 20–50 ng of DNA. The samples were fragmented and adapter sequences were added. These adapters were used in a limited-cycle PCR in which the material was supplemented with unique indices. The ultimate concentration of the libraries followed the library preparation. After library preparation, the libraries’ final concentrations were assessed using the Life Technologies Qubit^®^ dsDNA HS Assay Kit, and the average library size was estimated using the Agilent 2100 Bioanalyzer (Agilent Technologies, Santa Clara, CA, USA). After that, the libraries were pooled at 0.6 nM equimolar ratios and sequenced at paired end for 300 cycles on the NovaSeq 6000 system (Illumina, San Diego, CA, USA).

### 4.4. Metagenomics Data Annotation and Data Analysis

The sequences were uploaded on an MG-RAST server [45] at https://www.mg-rast.org (accessed on 24 August 2021). The quality control steps involve dereplication, which removes artificial sequences, ambiguous base-filtering, and host species-specific sequences. After the quality control process, annotation of sequences was performed using the BLAT algorithm [46]. This BLAST-like alignment tool was used against the M5nr database, which results in non-redundant alignment of several databases [47]. Taxonomic classification was carried out with the use of RDP database, while functional categories from level 1, level 2, and level 3 were assigned using the SEED subsystem database using default settings. Sequences that failed annotation were discarded and no further analysis was performed on them. Unclassified reads were maintained for statistical purposes while the functional table was constructed, abundances were then transformed into percentages, and the functional table was assembled according to each functional level. The raw sequences have been deposited with NCBI under the BioProject accession number PRJNA763981.

The environmental variables that determine the microbial composition were obtained using forward type CCA (canonical correspondence analysis). The differences in soil physicochemical properties between rhizosphere soil and bulk soil were calculated using ANOVA (a one-way analysis of variance) using Tukey’s pairwise comparison test. The Shannon, Simpsons, and the Pielou evenness of all samples were determined as a measure of diversity indices of each sample. The Kruskal–Wallis test was used to compare the resulting indices of the samples. Monte Carlo permutation test using 9999 random permutations was used to determine the test of significance. PAST version 3.20 [48] was used for statistical analysis and to determine the differences in the community composition within the same sample group. PCoA (principal coordinate analysis) based on the Euclidean distance matrix with one-way analysis of similarities (ANOSIM) through 9999 permutations were used to calculate the beta diversity variations. A shiny heat map with z-score was used to draw the heat maps [49]. The variable data used for CCA analysis was obtained from the environmental variables as shown in Table 1. Finally, CCA, PCoA, and PCA were plotted with CANOCO software [50].

## 5. Conclusions

Shotgun sequencing provides information about the functional roles of different microbial populations in the rhizosphere in fostering plant development and health. This study revealed the functions of all microbiota in the soil attached to the root part of soybean and the bulk soil. The pathways with the highest relative abundance at level 1 are carbohydrate metabolism, clustering-based subsystems (pathway with no established function), and amino acids and derivatives. It is anticipated that the microbial communities in these soils will assist plant growth, development, and survival in their diverse habitats due to the availability of these services in the soils. We also found many metabolic pathways, including those for sulphur, nitrogen, and secondary metabolism. The results of this investigation showed that the chemical properties of the soils are responsible for controlling the microbial functional diversity in the rhizosphere and bulk soils. The existence of these functional categories connected to many biological processes explains how the microorganisms respond to and adapt to their microenvironment and how their metabolic abilities can improve the growth and development of soybean.

## Figures and Tables

**Table 1:** The physical and chemical properties of the rhizosphere and bulk soil samples.

Sample	AA	AB	CA	BC
Sand (%)	88.00 ± 1.53 ^a^	86.00 ± 2.00 ^a,b^	88.30 ± 0.58 ^a^	84.00 ± 1.00 ^b^
Silt (%)	2.00 ± 1.00 ^a^	4.00 ± 1.00 ^a^	2.00 ± 1.00 ^a^	2.00 ± 1.00 ^a^
Clay (%)	10.00 ± 2.00 ^b^	10.00 ± 1.00 ^b^	10.00 ± 1.00 ^b^	14.00 ± 2.00 ^a^
pH	6.94 ± 0.04 ^a^	6.80 ± 0.03 ^b^	6.58 ± 0.03 ^c^	6.63 ± 0.01 ^c^
S (mg/kg)	543 ± 3.00 ^b^	563 ± 0.01 ^a^	501 ± 1.00 ^c^	496.3 ± 0.58 ^d^
Org C (%)	0.36 ± 0.03 ^b^	0.24 ± 0.02 ^d^	0.30 ± 0.02 ^c^	0.63 ± 0.01 ^a^
Org M (%)	1.62 ± 0.02 ^b^	1.44 ± 0.01 ^c^	1.40 ± 0.02 ^c^	2.66 ± 0.01 ^a^
P (mg/kg)	46.68 ± 0.01 ^b^	41.57 ± 0.03 ^c^	48.59 ± 0.01 ^a^	7.40 ± 0.01 ^d^
K (mg/kg)	81.46 ± 0.02 ^d^	93.14 ± 0.02 ^c^	97.48 ± 0.02 ^b^	106.58 ± 0.03 ^a^
Na (cmol(+)/kg	10.23 ± 0.03 ^a^	9.74 ± 0.04 ^b^	8.49 ± 0.00 ^c^	8.52 ± 0.03 ^c^
N-NO_3_^−^ (mg/kg)	2.29 ± 0.03 ^b^	3.48 ± 0.01 ^a^	2.22 ± 0.01 ^c^	2.01 ± 0.01 ^d^
N-NH_4_^+^ (mg/kg)	9.83 ± 0.01 ^c^	12.64 ± 0.03 ^a^	10.33 ± 0.01 ^b^	5.90 ± 0.01 ^d^
Total C (%)	0.38 ± 0.005 ^b^	0.26 ± 0.005 ^d^	0.32 ± 0.00 ^c^	0.64 ± 0.00 ^a^
Total N (%)	0.04 ± 0.002 ^b^	0.03 ± 0.003 ^d^	0.03 ± 0.00 ^c^	0.06 ± 0.00 ^a^

Values are triplicate mean ± standard error. Means with the same letter within the same row are not significantly different at *p* > 0.05. Samples AA, AB, and CA are rhizosphere soils while BC is bulk soil.

**Table 2:** Diversity indices of functional categories in both the rhizosphere and bulk soil samples.

Diversity	Sample AA	Sample AB	Sample CA	Sample BC
Shannon	1.851	1.947	1.815	1.763
Simpson	0.6712	0.7152	0.6652	0.6408
Evenness	0.1027	0.113	0.08902	0.09257

AA, AB, and CA are rhizosphere soil samples while BC is a bulk soil sample.

**Table 3:** Selection of variables in the environment that best explain the differences in functional composition of the microbes’ habitat using canonical correspondence analysis.

Name	Explain%	Contribution%	Pseudo-F	*p*-Value
Total N	30.8	30.8	0.90	0.91
P	64.3	64.3	13.2	0.718
K	4.9	4.9	<0.1	1

## Data Availability

The raw sequences have been deposited with NCBI under the BioProject accession number PRJNA763981.

## Supplementary Materials

The supporting information can be downloaded at: https://www.mdpi.com/article/10.3390/ijms23169422/s1.
