# Supplementary material for: Functional Diversity of Microbial Communities in the Soybean (Glycine max L.) Rhizosphere from Free State, South Africa
Source: Int J Mol Sci. 2022 Aug 20;23(16):9422. doi: 10.3390/ijms23169422 (PMC9409019; doi:10.3390/ijms23169422)
Supplement: Supplementary file 1 [file ijms-23-09422-s001.zip › ijms-1829058-supplementary.pdf]

Supplementary material

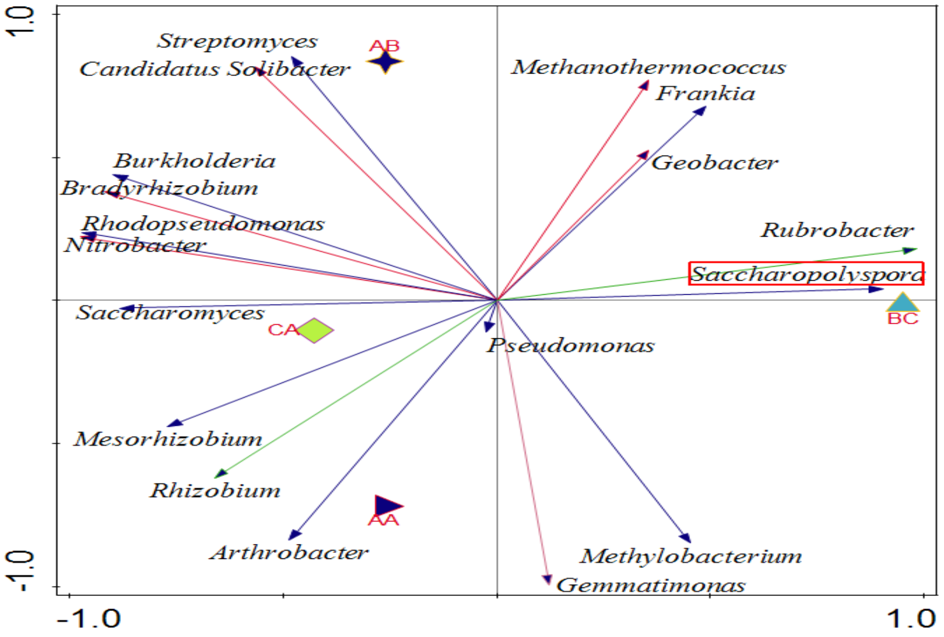

Figure S1. PCA analysis of the distribution of the genera across the sampling sites. AA, AB, and CA are rhizosphere soil samples while BC is bulk soil sample.

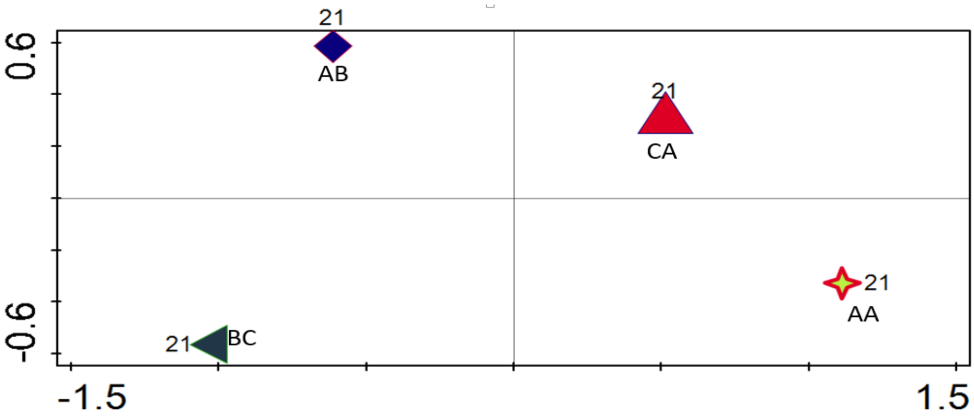

Figure S2. PCoA (Principal coordinate analysis) of functional categories of microbial communities in the rhizosphere and bulk soil samples. AA, AB, and CA are rhizosphere soil samples while BC is bulk soil sample.

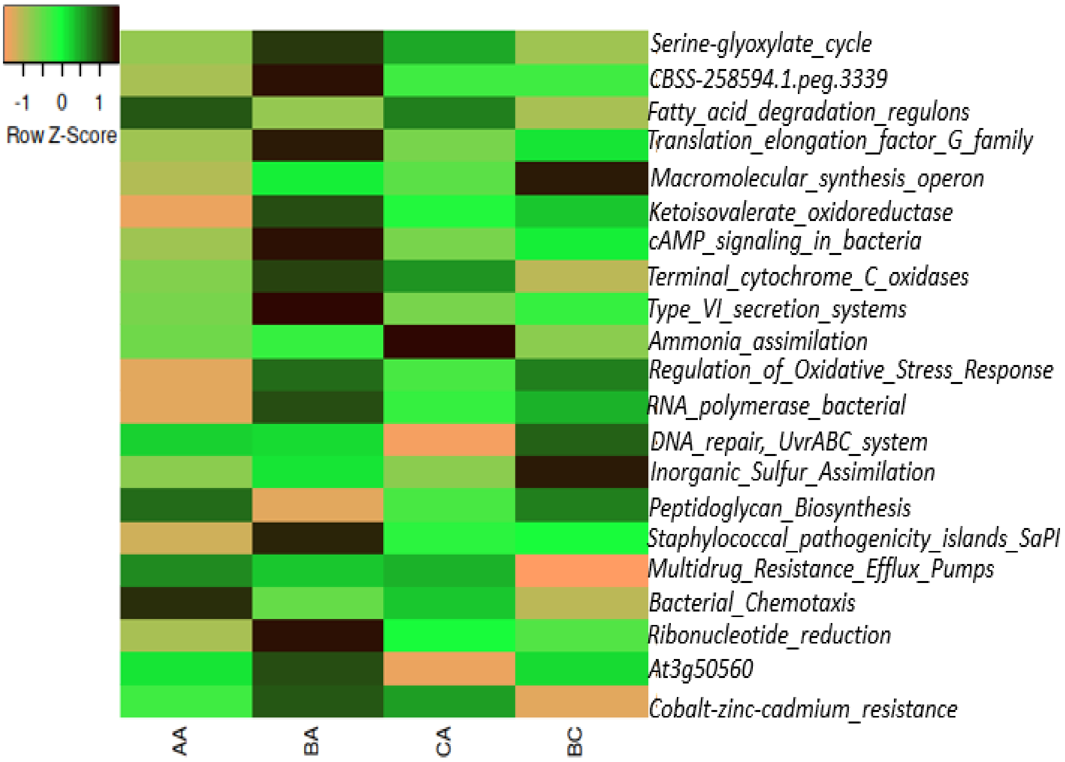

Figure S3. Pathway of selected pathways at level 3 susbsystem. The bar shows the saturated color gradient base on the relative abundance in Z-score which was gotten from metabolic pathways of the microbiomes in that habitat. AA, AB, and CA are rhizosphere soil samples while BC is bulk soil sample.

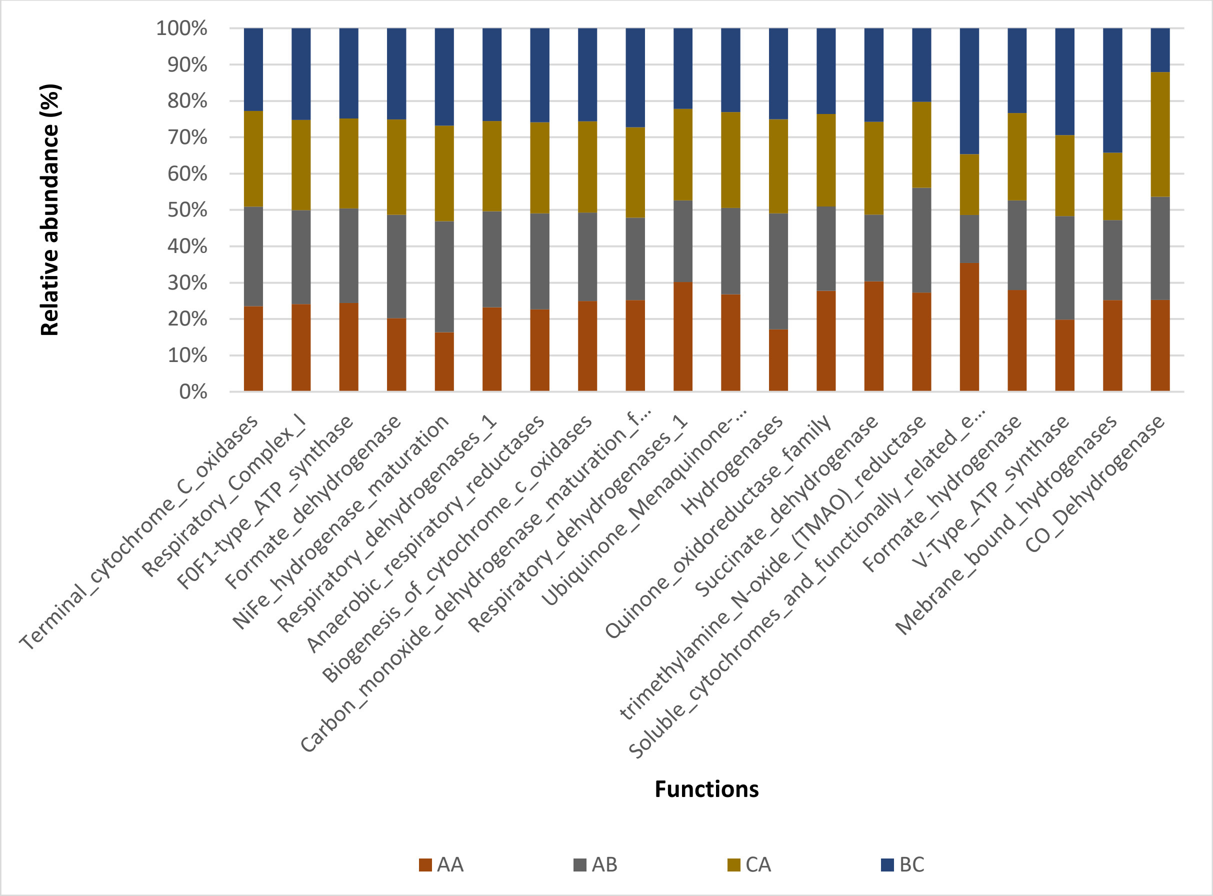

Figure S4: Cell metabolism (respiration) pathway at level 3. AA, AB, and CA are rhizosphere soil samples while BC is bulk soil sample.

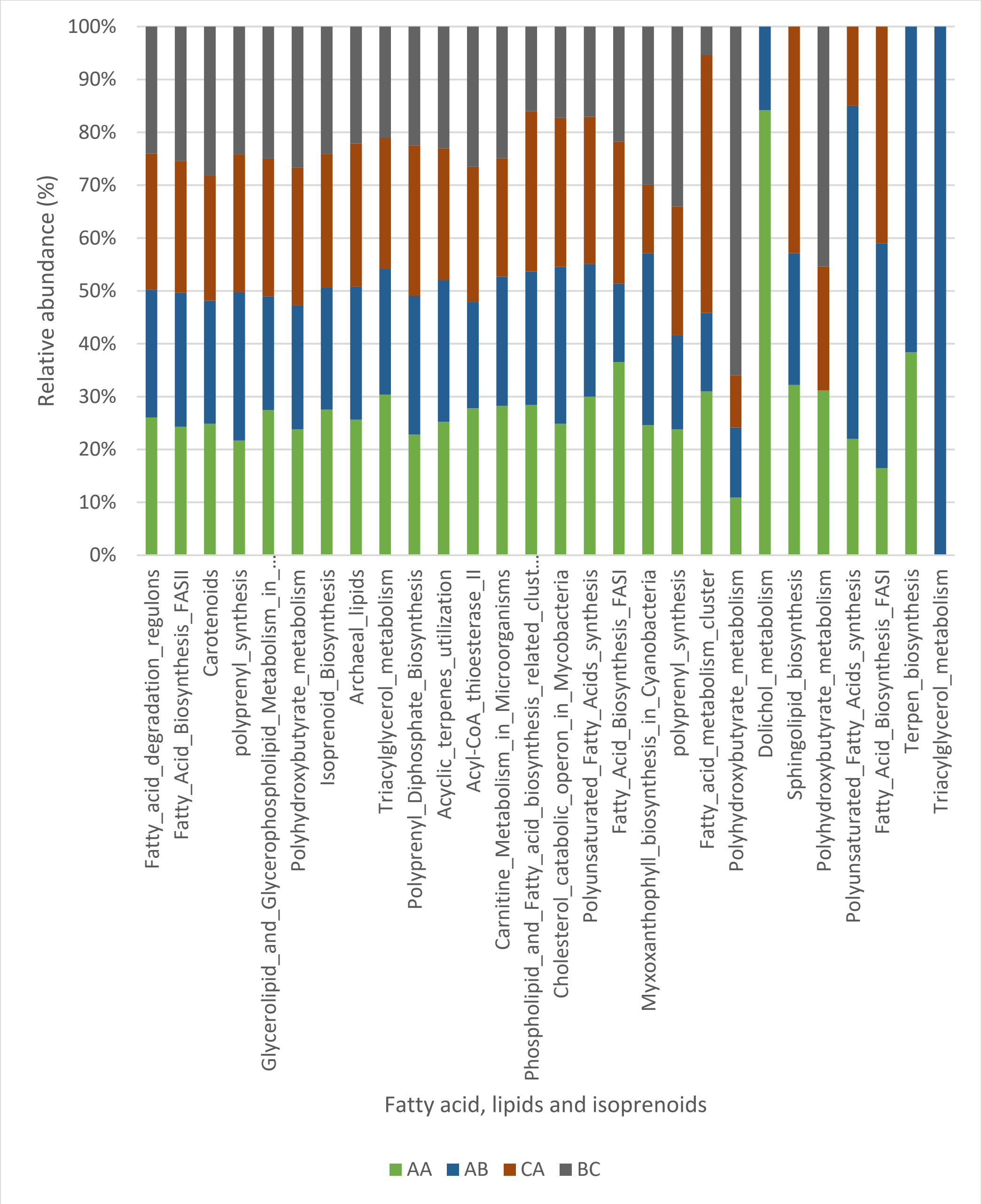

Figure S5: Pathway for selected fatty acid, lipids and isoprenoids metabolism. AA, AB, and CA are rhizosphere soil samples while BC is bulk soil sample.

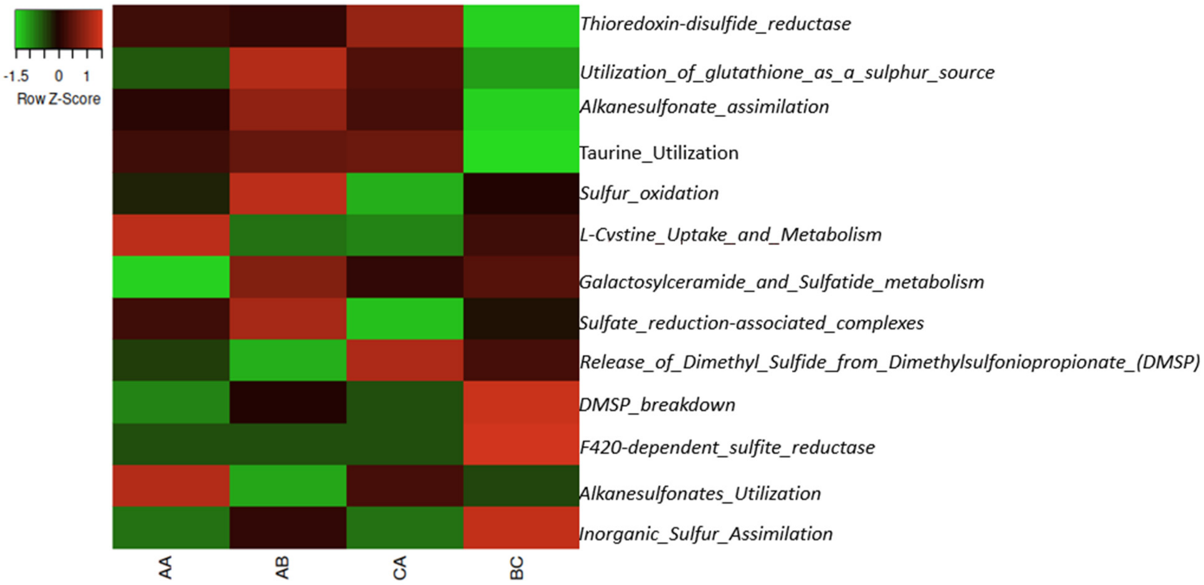

Figure S6: The sulfur metabolic pathway at subsystem level 3. The bar shows the saturated color gradient base on the relative abundance in Z-score which was gotten from metabolic pathways of the microbiomes in that habitat. AA, AB, and CA are rhizosphere soil samples while BC is bulk soil sample.

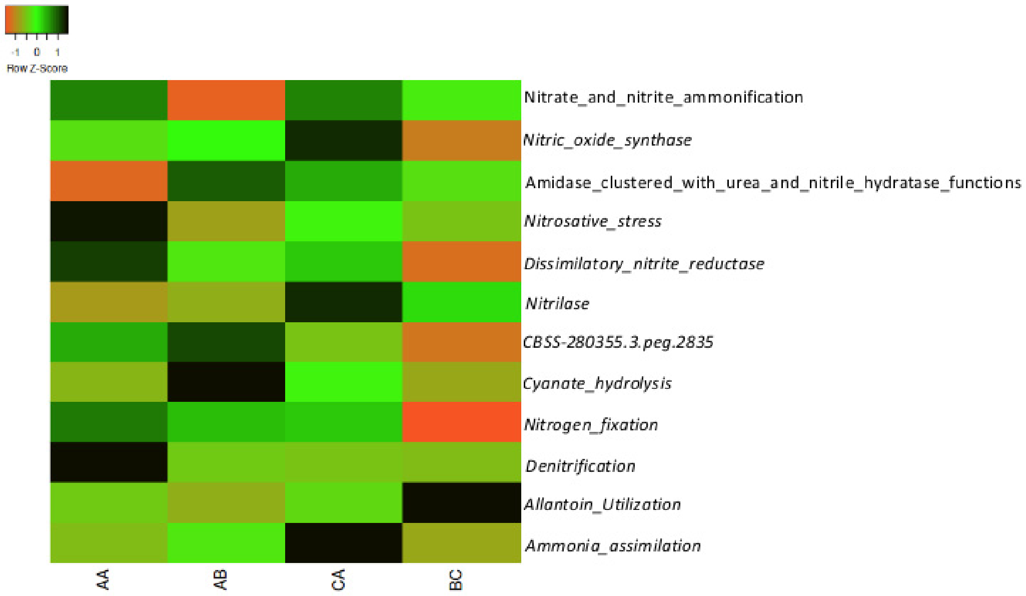

Figure S7; Pathway for nitrogen metabolism at level 3. The bar shows the saturated color gradient base on the relative abundance in Z-score which was gotten from metabolic pathways of the microbiomes in that habitat. AA, AB, and CA are rhizosphere soil samples while BC is bulk soil sample.

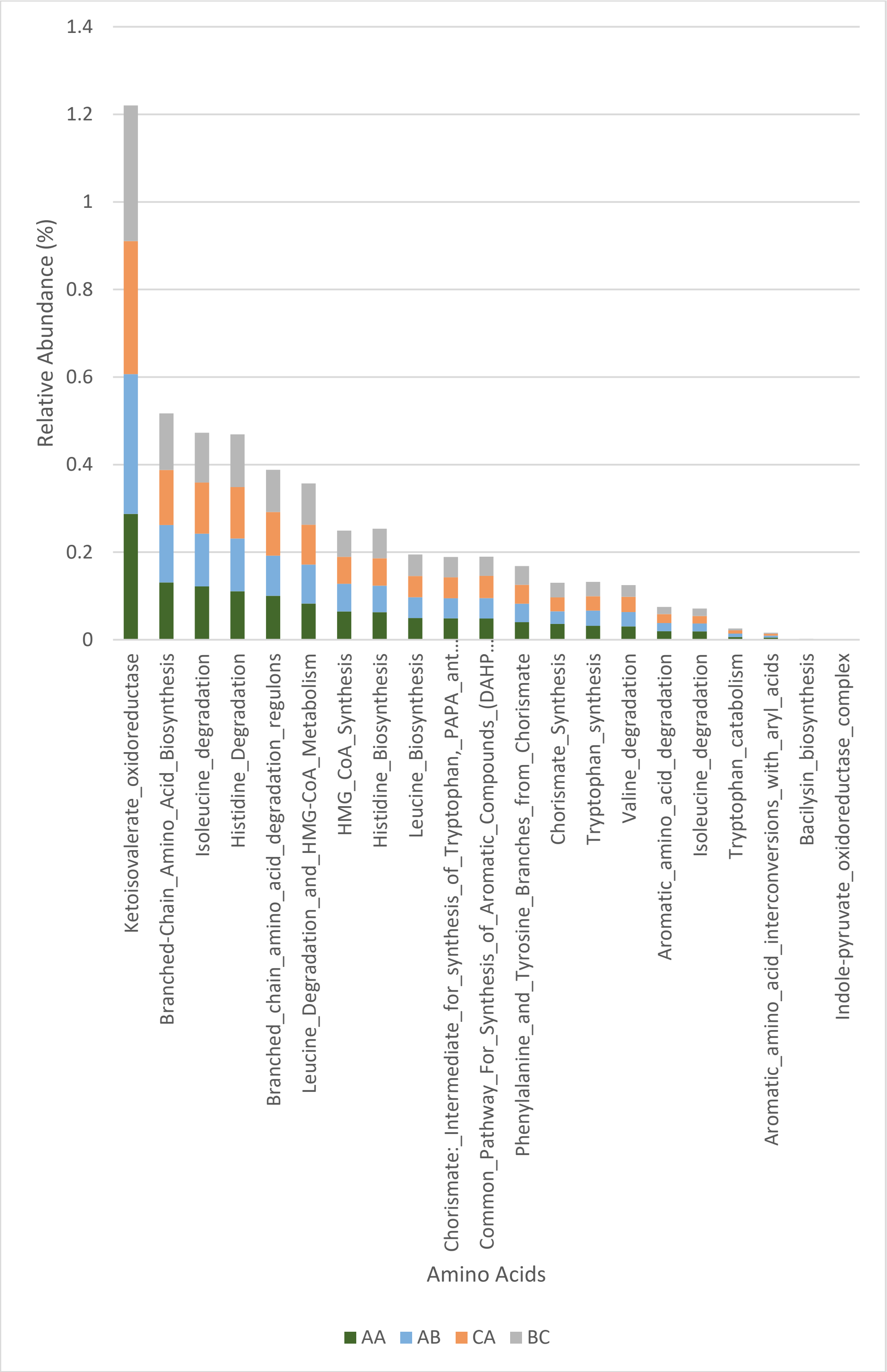

Figure S8: Bar-chart showing the pathway for histidine, aromatic and branched amino acids. AA, AB, and CA are rhizosphere soil samples while BC is bulk soil sample.
